# Pharmacogenetic Testing: A Tool for Personalized Drug Therapy Optimization

**DOI:** 10.3390/pharmaceutics12121240

**Published:** 2020-12-19

**Authors:** Kristina A. Malsagova, Tatyana V. Butkova, Arthur T. Kopylov, Alexander A. Izotov, Natalia V. Potoldykova, Dmitry V. Enikeev, Vagarshak Grigoryan, Alexander Tarasov, Alexander A. Stepanov, Anna L. Kaysheva

**Affiliations:** 1 Biobanking Group, Branch of Institute of Biomedical Chemistry “Scientific and Education Center”, 109028 Moscow, Russia; t.butkova@gmail.com (T.V.B.); a.t.kopylov@gmail.com (A.T.K.); farmsale@yandex.ru (A.A.I.); aleks.a.stepanov@gmail.com (A.A.S.); kaysheva1@gmail.com (A.L.K.); 2 Institute of Urology and Reproductive Health, Sechenov University, 119992 Moscow, Russia; potoldykovanv@gmail.com (N.V.P.); enikeev_dv@mail.ru (D.V.E.); nii-uronephrology@yandex.ru (V.G.); 3 Institute of Linguistics and Intercultural Communication, Sechenov University, 119992 Moscow, Russia; alexgarmisch@yandex.ru

**Keywords:** pharmacogenetics, pharmacogenetic test, personalized medicine, genetic polymorphism

## Abstract

Pharmacogenomics is a study of how the genome background is associated with drug resistance and how therapy strategy can be modified for a certain person to achieve benefit. The pharmacogenomics (PGx) testing becomes of great opportunity for physicians to make the proper decision regarding each non-trivial patient that does not respond to therapy. Although pharmacogenomics has become of growing interest to the healthcare market during the past five to ten years the exact mechanisms linking the genetic polymorphisms and observable responses to drug therapy are not always clear. Therefore, the success of PGx testing depends on the physician’s ability to understand the obtained results in a standardized way for each particular patient. The review aims to lead the reader through the general conception of PGx and related issues of PGx testing efficiency, personal data security, and health safety at a current clinical level.

## 1. Introduction

P4-medicine represents an actively developing field of modern medical science. The P4 conception is based on a personalized approach to human health (Personalization, Prediction, Prevention, and Participation). Modern diagnostic kits allow the identification of human metabolic characteristics at the molecular level, thus enabling the revelation of a personal, genetically determined predisposition to a disease or certain metabolic disorders in particular individuals [1]. Personalized medicine involves drug therapy to improve the patient’s condition and minimize any adverse effects, thus increasing the quality of life at both the individual and socioeconomic levels.

Pharmacogenetics goes back to 1959 when Vogel coined this term to designate severe adverse drug reactions in a small number of patients reported by the pharmacologists [2]. The adverse reactions, which followed the administration of primaquine, succinylcholine, and isoniazid, were anemia, apnea, and peripheral neuropathy, respectively [3,4].

Pharmacogenetics is purposed to study the response to the drug therapy depending on the genetic background. Response to drugs is frequently governed by genes encoding drug-metabolizing proteins, thus, regulating drug transformation, pharmacokinetics, and pharmacodynamics [5].

PGx may support the investigation of the effect of vitamins [6,7] and additives/supplements and, to some degree [8], homeopathic preparations. However, there is no strong evidence regarding the interaction between genes and vitamins/supplements/homeopathies.

The majority of the related assays in PGx are erroneous or misinterpreted due to biases in the design of the experiment, small sampling, small size of the population, and insufficient time of observation. So far, it is obligatory to provide more correct experiments and researches to observe the suggested effects, otherwise, most of the discussion about the possible influence of non-pharmacological compounds on the genome could be considered doubtful.

In clinical practice, a physician follows the national standards of specialized medical care, based on the evidence from fundamental research and clinical trials of drugs. However, to a greater extent, the therapy process remains a creative task [9].

In addition to adverse drug reactions, the body can demonstrate immunity and/or just partial response to the treatment [10]. Despite the underlying causes of drug resistance remain unclear; however, one can suppose that this is connected with genetic factors. Moreover, aside from the predisposition to diseases, various body metabolic functions are also determined genetically. Namely, genetic variations probably determine the rates of synthesis and decay of multiple biomolecules in the body, the effect of pharmaceuticals, the metabolism of nutrients, etc. [11]. However, answers to these questions have not yet been received.

Nevertheless, genetic testing is slowly finding its niche in drug therapy selection—this process is followed by improving care to a widening range of patients. Pharmacogenomic Biomarkers in Drug Labeling Food and Drug Administration (FDA) provides data about 297 drugs for 100 molecular biomarkers (www.fda.gov/drugs/science-and-research-drugs/table-pharmacogenomic-biomarkers-drug-labeling). Several companies offer genetic testing for adverse drug reactions in patients.

The main goal of this paper is to review current and the latest (up to the latest five years) achievement and progression in PGx relevant to human healthcare and personalized medicine (excluding animal models). The review summarizes general information about pharmacogenomics and trends based on the current level of PGx testing and clinical application for the past decade. The review aims to outline the main approaches used in PGx and provide a brief overview of the related issues and criticism of shortcomings

## 2. Pharmacogenetic Studies of Drugs

The success of the “Human Genome” project gave impetus to molecular medicine, representing a new branch of medicine focused on the genetic marker panel. Genetic markers represent point nucleotide polymorphisms, which are individual for each person, and reflect his/her personal characteristics.

Even though the growing amount of available data on single nucleotide polymorphisms (SNPs) and other types of genetic mutations makes a significant contribution to the revelation of genome structural variability, the functional importance of these pharmacogenetic variations remains unclear.

Gene variants–alleles- are designated with an “asterisk” followed by a number (e.g., * 1, * 5, * 13) and include one or more SNPs, which are inherited together. Alleles have various levels of activity identified by number, where * 1 (haplotype) denotes a “wild type” or lack of any detected variation [12].

**Figure 1:**
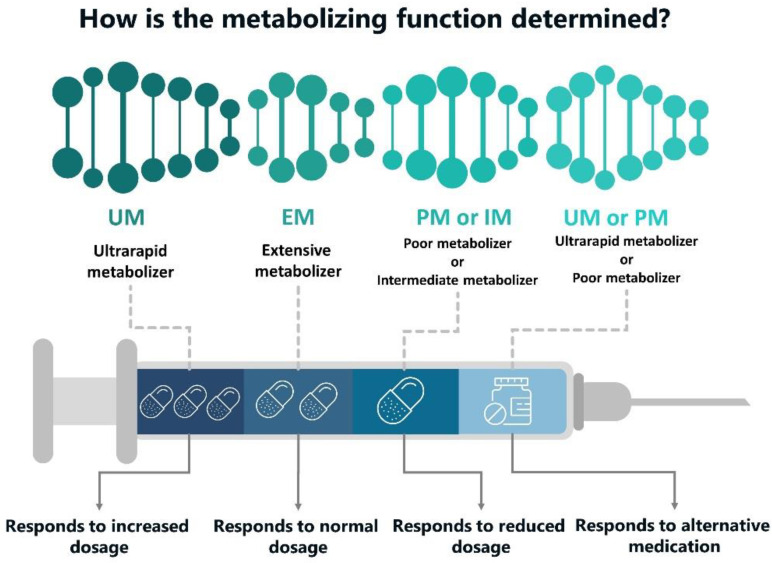
Genetic variation in metabolic phenotype. Depending on the pharmacogenomics (PGx) testing of genes encoding enzymes that are involved in the drug transport and transformation activity, the examined person can be attributed to either of the defined phenotype (ultra-fast metabolizers (UM), extensive metabolizers (EM), poor metabolism (PM), or intermediate metabolizers (IM)) which, in turn, indicates a personal response to dosage and the certain medication.

Pairs of these stellate alleles (diplotypes) are subdivided into phenotypes based on their enzymatic activity:

- poor metabolism (PM): a type of alleles that carry the mutated gene(s) encoding important metabolizing enzyme that participates in drug transformation and exhibition of drug activity. Such mutations cause the synthesis of either an insufficient amount of enzyme or produce its inactive gene product which entails decreasing of enzymatic activity and even complete loss of activity. They are much slower to eliminate various drugs metabolized by the same enzyme. Therefore, the patient runs a risk to reach a high plasma concentration of the drug, causing dose-dependent adverse effects. In this regard, slow metabolizers require a careful drug dose selection:
- Extensive metabolizers (EM): they provide a regular rate of drug biotransformation. They usually have two active allelic genes or one functional and one partially active allele;
- Intermediate metabolizers (IM): heterozygous carriers of the mutation (with an autosomal recessive inheritance). To achieve an optimal therapeutic effect, they may require a lower pharmacological dosage than the usual one;
- Ultra-fast metabolizers (UM): they are characterized with an increased gene expression—owing to the presence of three or more functional alleles following the duplication or multiple duplications of a functional allele (e.g., duplication of the CYP2D6 gene). Ultra-fast metabolizers may require a higher drug dose for an optimal effect (Figure 1).

UM and PM metabolizers represent the groups connected with the most significant risk of therapy ineffectiveness or adverse effects [13].

**Figure 2:**
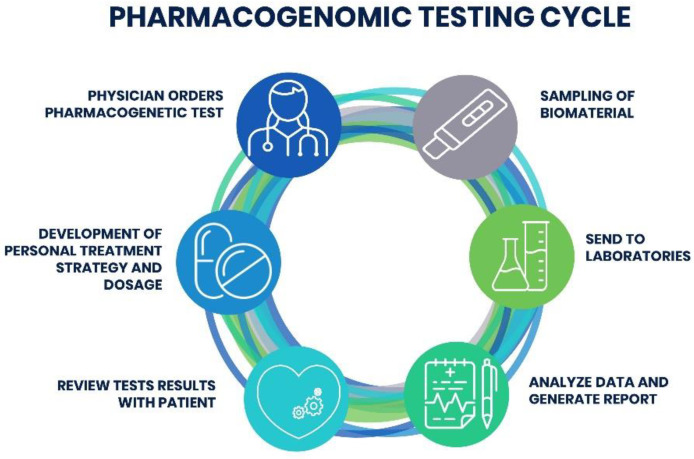
Schematic representation of PGx testing steps. Initially, the physician orders the PGx testing to the curated patient. The test is usually performed on saliva or peripheral blood and requires a small amount of biomaterial, and does not require special preparation for the test. After the testing, the physician reviews the obtained report and discuss it with the curated patient to find out whether certain medication can be effective and what is the best dosage for the treatment therapy. The results may also include the prediction of possible side-effect from the prescribed medication.

Even though clinical sites and laboratory centers have an individual approach to the need for PGx testing and the workflow, there are four main stages. These steps include: (1) patient identification, (2) taking the biomaterial for the PGx testing, (3) sending the biomaterial to the laboratory to perform the selected PGx test, (4) analyzing the obtained results, and (5) review the results by a professional physician together with a curated patient to, (6) eventually, elaborate the treatment strategy (Figure 2).

These steps are not meant to be exhaustive or set out in guidelines. Hospitals can adapt the steps outlined to their individual practice structure, patient needs, and clinical priorities.

Planning a personalized medicine study design depends on the goal. The first stage of the planning involves searching for candidate genes-genes whose transcription products affect the pharmacokinetics or pharmacodynamics of drugs. Typically, this stage includes a literature review. Most of the candidate genes are known and are being actively studied. If there is no data in the literature on the effect of genetic polymorphisms on the investigated drug’s efficacy and safety, it is necessary to conduct its research.

### 2.1. Comparative Cohort Study with Posterior Analysis

Posterior research is the most common pharmacogenetic analysis design where patients with a specific nosology are selected based on the studied drug’s indication. The control group is either the “placebo” group or the group provided therapy with an alternative medication. Simultaneously, the parameters of the effectiveness and safety of treatment in the comparison groups are evaluated. The study ends with an analysis of the association of the patient’s genotype for the studied polymorphic marker with the results of therapy. It is possible to establish a possible association between the presence of a polymorphic gene variant and the impact of pharmacotherapy. The genotyping of a sample of the already completed study is performed to implement this design. The main disadvantage is that it is impossible to influence the number of carriers of the polymorphic variant, which may be insufficient for statistical analysis [14,15].

### 2.2. Comparative Cohort Study with Genotyping of Participants before Inclusion

The study can be carried out in two versions:

1. Posterior analysis, in which genotyping is carried out before inclusion in the study to form subgroups of equal number with the “wild” genotype and polymorphic variant and does not affect the appointment of pharmacotherapy;
2. The assignment of pharmacotherapy is carried out depending on genotype to determine whether a study drug actually has an advantage in this patient population over an alternative (or placebo) [14,16].

### 2.3. Comparative Study of the Pharmacogenetic Approach

A comparative study of two approaches, traditional and pharmacogenetic, is conducted if rigorous evidence about the influence of genetic polymorphisms on a particular drug’s efficacy and safety exists. This is the final stage before the introduction of pharmacogenetic testing into clinical practice. The population includes patients with indications for the study drug. The drug dose selection is carried out based on the results of pharmacogenetic testing, and the traditional selection of the dose of the given drug serves as a control. As a result of the study, the advantages of a personalized approach are assessed in comparison with an empirical one [14]. This study alone is not enough. To achieve the highest level of evidence, a meta-analysis of several studies with this design is required.

It is important to determine the mechanism underlying the variability of drug response and drug efficiency, to establish the starting point that can support the identification of genes that produce the necessary pharmacogenetic effect. Therefore, many instances in pharmacogenetics relate to the personal assimilation, metabolism, or elimination of a drug. Other features contributing to the variable drug responses include distinctions in drug target molecules or disease pathways. In some cases, variants in several genes are implicated (“combinatorial pharmacogenetics”) in the determination of variable response. Recently, searches for previously unexpected relationships between phenotypes and thousands of common polymorphic sites in the genome (an unbiased approach) have been utilized to address the problem of variable drug action [17].

## 3. Prospects for the Introduction of a Pharmacogenetic Test into the Clinic

Nowadays, personalized medicine becomes more and more important [18]. Ongoing clinical trials can result in the introduction of pharmacogenetic testing into practice. This may accelerate the approval of distinct medication with no obligation to be tested on a PGx matter, which makes their market entry faster and more cost-effective. Typically, genomic information related to individual patients is made available to those who prescribe therapy. By 2017, The UK planned to perform the sequencing of 100,000 genomes of cancer patients and patients suffering from occasional or most dangerous infectious diseases (HIV, hepatitis C, tuberculosis), and to provide the pharmacogenetic information about the patients admitted in the study by the National Health Service [19]. Company 23andMe (Sunnyvale, CA, USA) (www.23andme.com) also provides pharmacogenetic information to guide the treatment directly upon customer request [20]. The FDA approved the first pharmacogenetic test in 2005. That was AmpliChip CYP450 test system manufactured by Affymetrix (Santa Clara, CA, USA) and Third Wave Technologies (Madison, WI, USA) Invader UGT1A1 (UDP-glucuronosyltransferase) Molecular Assay. The approval procedure was endorsed by clinicians, general healthcare systems, insurance companies, and concerned patients to determine the best way to integrate these tests into clinical practice [21]. At the same time, the critical questions raised were as to whom the tests should be applied and what are the most appropriate circumstances for their application, what evidence is required for the application of these tests, and how the results of the tests must be stored in electronic health data depositaries [22]? Haga and Kantor [23] reviewed laboratories, which offer clinical PGx testing in the United States. Of the 111 reviewed laboratories, 76 offered PGx testing services. Of these laboratories, 31 laboratories offered tests for only specific genes; 30 laboratories offered tests for multiple genes, while only 15 laboratories offered both types of tests. A total of 45 laboratories offered 114 multigene panel tests that cover 295 genes. However, no clinical guidelines were available for most of these tests [23]. In the industry, there is a trend towards multiplex tests intended to detect polymorphisms in a large number of genes. In 2005, the FDA-approved AmpliChip test (Roche, Basel, Switzerland) was designed to analyze two genes [24]. In 2010, Affymetrix introduced the DMET chip to diagnose 225 genes; the latter number is now expanded to 231 genes [23,25]. In 2012, researchers from Stanford University and the University of Florida developed a panel containing an SNP array of 120 genes including 25 genes responsible for drug metabolism and 12 drug carrier genes [26]. In 2014, the PGRN-Seq capture test for the analysis of 84 pharmacogens was developed [27]. The variety of gene panels is not limited to the examples mentioned above. Other types of pharmacological tests are available or are under development [28,29].

Currently, the clinical relevance of multigene panels mainly depends on a few well-studied and classical genes. Using the 84 gene PGRN-Seq capture panel, the examination of only five genes among ca. 5000 patients indicated that 99% of tested patients carried at least one clinically valid variant or one known variant relevant to decide about their treatment [30]. However, the clinical significance of large multiplex panels can mainly be determined by a certain task to be solved. For instance, PGx testing can be arranged as an immediate decision on treatment based on a panel of genes with a high level of evidence for a particular drug. However, the most common type of multigenic complex panel PGx test without specific clinical indications can be performed for a forward-looking patient. Such tests can be warranted since alternative drugs can be further used, and the clinical relevance of the data can increase with time. Despite the absence of consensus on the preventive PGx testing [31,32], many healthcare organizations implemented such testing programs to obtain valuable information regarding clinical validity and usefulness [27,33].

### 3.1. Audience for PGx Testing

Currently, PGx tests exist in various areas of medicine, including, but not limited to, psychiatry, cardiology, anesthesia, and oncology. Some clinical guidelines for PGx tests are accessible for the prediction of tricyclic antidepressants (TCAs) and selective serotonin reuptake inhibitor (SSRI) efficacy based on CYP2D6 and CYP2C19 activity [34,35]. Recent studies revealed reduced adverse effects and improved scores in depressed patients after PGx-based antidepressant therapy [36]. PGx testing is particularly attractive given the time frame required. For instance, the estimation of the complete therapeutic response to SSRIs can require 4 to 6 weeks [37]. The patient and the healthcare provider can spend several months adjusting the dose and/or prescribing new medications before it becomes clear that the therapy does not lead to the therapeutic effect. PGx testing can allow a physician to determine the best drug for a given situation in a much shorter time.

PGx testing applicability, to a certain extent, depends on the intensity of potential adverse reactions to the drug. For instance, Abacavir, used for HIV treatment, can produce severe cutaneous adverse reactions (SCAR) [38]. Generally, the risk is low, but HLA-B*57:01 variation is related to much more pronounced SCAR after Abacavir intake. Thus, this drug is contraindicated for HLA-B*57:01- positive patients [38].

The use of PGx testing is relevant to the selection of the Warfarin dose. Intake of dietary vitamin K, health and social conditions, and genetic variations were also found to affect Warfarin therapy [39]. Changes in CYP2C9 can disrupt the metabolism of Warfarin, and alterations in VKORC1 (vitamin K epoxide reductase) can increase the drug susceptibility of a patient [38,39].

The use of codeine was limited to adult patients after the evidence of a risk of increased adverse effects in pediatric patients. The study revealed adverse reactions in infants whose breastfeeding mothers underwent codeine therapy [40]. These reactions resulted from codeine conversion to morphine, performed mainly by CYP2D6 protein [41]. Similar reactions can occur with other CYP2D6-mediated pain relievers—such as tramadol, oxycodone, and hydrocodone [42].

The high interest of patients in PGx testing was revealed [43,44]. The patients are particularly interested in the possibility of using recommendations based on PGx test results to reduce adverse drug effects and to choose proper therapy [45]. However, the cost of the tests, the insurance coverage, and the availability of testing results represent the limitations for PGx testing [46,47]. Some questions are still relayed uncertain after PGx testing. Thus, patients should be appropriately informed about the capabilities of PGx testing [48].

For this reason, one should understand that PGx testing allows the identification of (1) drugs with an increased risk of causing adverse effects, (2) drugs with a narrow therapeutic index. Besides that, PGx testing can reduce the set of drugs for therapy and predict the drug dosage [48,49]. At the same time, PGx testing will not be efficient for predicting: (1) occurrence of all possible adverse reactions with a drug, (2) the risk of a specific adverse effect for all drugs, 3) the risk of occurrence of complications [49].

The PGx testing provides an opportunity in decision making of whether the chosen medication and treatment strategy is of advantage and gives the proper results over the expectations based on the obtained profile of patients. That is exactly what opens the door for personalized medicine, that is what should happen when a person has a choice: to be healed but not at a cost of health deterioration, not in awaiting while inappropriate drug boosts dire consequences instead of a satisfying outcome.

### 3.2. Resources in the Pharmacogenetic Sector

Due to the ever-changing nature of genetic medicine, one should be aware of further changes in testing guidelines or results interpretation. This task can be simplified with the use of currently available Internet resources (Table 1).

Besides, there are also fewer known resources that merit attention. Among them, the Mayo Clinic portal that published numerous “AskMayoExpert” educational materials for both physicians and patients to enhance general knowledge and practice [54]. St. Jude Children’s Research Hospital allows tracking the website-integrated gene or drug information and implementation-specific publications and presentations [55]. Ubiquitous Pharmacogenomics (U-PGx) developed an e-learning platform to disseminate general knowledge of pharmacology, suitable for physicians and pharmacists (https://upgx.eu/) [56].

Support and development of these resources can be a valuable tool for studying the upcoming or less known pharmacogenetic interactions. Several organizations are currently providing integration with PGx programs and updating the current data [51,57].

### 3.3. Choice of PGx Testing

PGx testing can be carried out either as a single gene analysis or as a multiplex panel of ten or more genes. Early testing mostly involves analyzing multiple variants of the same gene, targeting the most common and most effective variants. Novel technologies significantly increased the number of genes and variations covered by a single test. Most PGx tests analyze a variety of clinically relevant SNPs. During the determination of the best test or panel for a patient (or a population), one should consider the therapeutic indication. For instance, testing for CYP2D6 and CYP2C19 is required for antidepressant therapy. In addition, it will also be useful to consider whether the patient would benefit from a PGx test for cardiovascular and pain-killing drugs [58]. A panel test can be more expensive than a single gene assay, but it ensures that co-prescribed drugs are also tested.

Panel PGx tests are heterogeneous and vary in volume and scope [59,60]. Most of the panels cover several of the best-studied and most potent genes. The panel can contain SNP combinations based on a literature review of prospective studies. A panel that includes many genes may not necessarily provide additional value to the patient, as not all options offer the same clinical relevance. Some variants can be extremely rare outside of certain populations but can be quite common within a certain group. For instance, the HLA-B * 15: 02 variant, associated with an increased risk of SCAR in patients prescribed carbamazepine, has an allele frequency of 0.04% in patients of European descent and 6.88% in patients of East Asian descent [61]. A panel can be more beneficial to the patient if it analyzes options that match their ethnic origin more closely.

One should keep in mind available alternatives or special analyses. Multiple copies of the CYP2D6 gene (e.g., duplications) occur in about 1 in 8 patients, and this number may be even higher in black and Asian patients [62]. Gene duplication can cause increased enzymatic activity and can be clinically relevant. However, not all panels can reveal the presence or degree of gene duplication.

In addition to the panel contents, one should consider such factors as the type of biomaterial (buccal smear, saliva, or blood, etc.) since the way of biomaterial sampling can pose a problem for the patient. The access to results and the methods of their obtaining also represent the factors that should be considered. Several panels provide nothing but raw genetic data, while others are fully integrated into the electronic health record and enable sophisticated clinical decision support systems (CDS). Finally, one should consider the potential cost of PGx testing. Patients vary in ways and means to pay for testing.

### 3.4. Interpreting PGx Test Results

When prescribing PGx testing, one should keep in mind that the result obtained represents only a part of the overall picture of the patient’s condition. Therefore, to determine the therapy risks and benefits, the PGx test results should be used and interpreted by taking into account the state of all the patient’s systems, concomitant medications, and current pathological conditions. When the “best” drug is identified with the PGx test, this does not necessarily mean that it should be used in therapy since the patient may have a history of severe adverse reactions to the drug. Conversely, the PGx test identification of an increased risk of therapeutic failure should not lead to drug discontinuation if the current therapy is effective. Different result structures can be used, depending on the gene and protein in question [63]. Some genes can be described in terms of metabolic activity, some by their general function, and others only as present or absent. Several PGx test results describe general gene function—such as SLCO1B1 (solute carrier organic anion transporter family member 1B1), associated with simvastatin; VKORC1 (vitamin K epoxide reductase complex subunit 1), associated with Warfarin; OPRM1 (opioid receptor Mu 1), associated with opioids [63]. Results for these genes can be reported as normal, intermediate, or low function. For instance, a “normal gene function” result indicates that no change in the patient’s dosage regimen is required. In other cases (decreased or poor function), the physician’s recommendations will be based on the information on a reduced functional activity (or complete inactivity) of the analyzed genes.

PGx test results can be “positive” or “negative” [57]. For instance, human leukocyte antigen (HLA) genes produce essential components of the immune system. Patients who are positive for HLA-B * 58: 01, run an increased risk of hypersensitivity to allopurinol; patients, who are positive for HLA-B * 15: 02, run an increased risk of SCAR with carbamazepine or oxcarbazepine [64,65].

The way the results are presented can vary considerably from one report to another. The results can be delivered either as raw genetic data or as the ultimate therapeutic recommendation. In the reports, a proprietary iconography can be used for the description of results. This iconography can use specific symbols to indicate patients in which an increased risk of adverse effects or therapy ineffectiveness is expected. Other reports can use a traffic light view with three main drug categories: green for normal risk, yellow for use with caution, and red for exclusion. Results displayed in any format can cause the provider to oversimplify the PGx test results, ignoring additional clinical considerations. 

**Figure 3:**
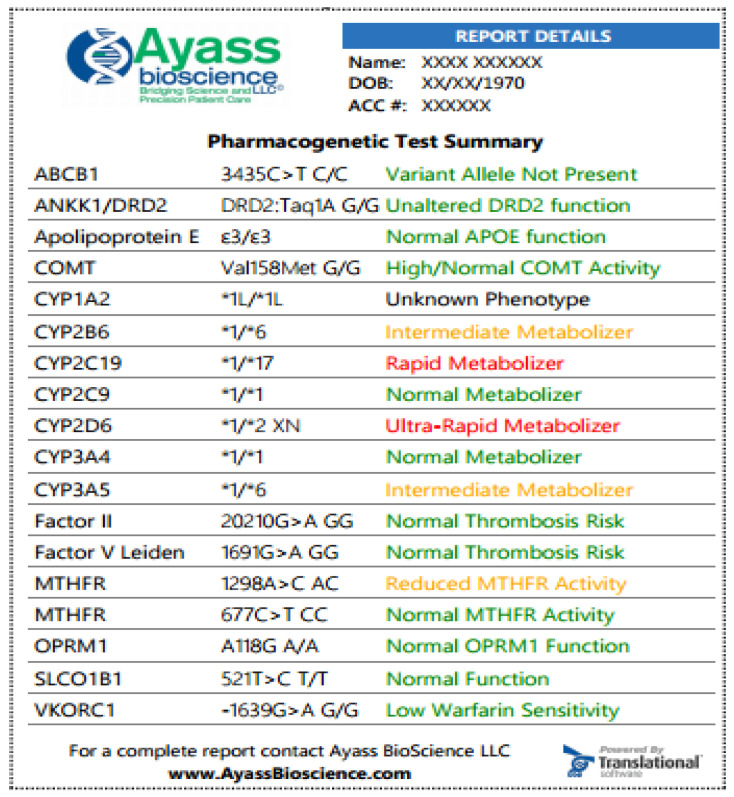
Samples of PGx test at AyassBioScience: The report is featured with color-coded information for easier navigation and attention. The greed color indicates that the medication can be prescribed according to standard regimens, and the risk for the indicated condition is not increased; yellow color—indicated that dosage adjusting is required, there is an increased vigilance or the patient has a moderate risk for the indicated condition; red color designates that medication has potentially reduced efficacy and increased toxicity or the patient has an increased risk for the indicated condition. In the exemplified results, the patient has a normal response to Apixaban (the drug is a substrate for the efflux transport proteins P-gp (ABCB1) and BCRP (ABCG2) and, possibly, decreased response to Bupropion. Bupropion is metabolized to its active metabolite hydroxybupropion by CYP2B6. This metabolite contributes to the therapeutic effects of bupropion when used as a smoking cessation agent or as an antidepressant. The patient has also increased response to Codeine, which is converted into its active metabolite morphine by CYP2D6. Since this patient is the ultra-rapid metabolizer (UM), a greatly increased morphine level is expected, and the patient is at high risk of toxicity when taking codeine.

Below (Figure 3) is an example of an abbreviated PGx test performed by AyassBioScience (Frisco, TX, USA). (the full report can be found at https://ayassbioscience.com/wp-content/uploads/2020/02/PGX-Medical-ManagementPrint-Fit-on-Page.pdf).

A variety of ways of presenting the information can be used in the report. Since each way is different, one should be careful to ensure a complete understanding of the meaning of each categorization [49].

### 3.5. Automation Tools for Integrating PGx Testing into the Clinic

Rapidly developing new technologies of DNA sequencing enabled quick and efficient identification of the genomic characteristics of organisms. The main result of the genomic and post-genomic technologies development was a significant expansion of the capabilities to study the genetic nature of a whole spectrum of human diseases. A genome-wide association study (GWAS) of clinical samples generated data on the genetic makeup featured for the specific groups (families or populations) to elaborate a personalized treatment approach. In this regard, to date, the research into the mechanisms of genetic predisposition to multifactorial diseases and the identification of specific genetic markers are of particular relevance. Such methods are widely used internationally and in Russia, where modern sequencing technologies are gradually introduced into medical research and medical practice to personify the treatment strategy.

Next-generation sequencing (NGS) is used for in-depth (multiple) reading of genetic material, which is necessary, for instance, for re-sequencing and assembly of new genomes (de novo), transcriptome, and epigenomic studies. This method allows one to reveal rare variations and to understand the genetic function better. However, the avalanche of new data will also make problems for researchers and clinicians, giving many “options of unknown importance” in the absence of clear indications [49]. Modifications of CDS are required to ensure the storage and use of new data architecture and new data availability programs. This, in turn, will identify significant opportunities in the coming years. Several healthcare systems use CDS tools to integrate PGx test data into clinical decision-making and provide information to end-users [66]. CDS systems can be used to administer high-risk drugs and provide automated recommendations indicating why certain modifications should be applied to a selected drug or dose.

The U-PGx PREPARE study developed solutions for sites with limited electronic health record infrastructure. The “Safety-Code” card is part of a mobile CDS, and with a quick response code, a medical professional is directed to a website with dosing recommendations customized for the patient [67]. This card also provides an overview of the most relevant PGx test results with a list of drugs with existing (known) recommendations [68].

Such CDS tools will be necessary, as PGx tests become more common due to the emergence of new results and test formats. One can also focus on developing patient-centered applications and portals, through which the patient can interact with his or her service providers and receive a consultation based on outcomes.

The increase in providers’ and patients’ awareness can stimulate the use of PGx testing. Consequently, laboratories will adjust the scope and type of available clinical PGx tests based on clinical requirements. The expected increase in the development of multigene panel PGx tests follows the advances in oncology, microbiology, and other fields. However, the proper clinical use of such tests appears to be more involved, requiring the support and participation of multiple interested parties.

## 4. Side Effects of Drugs and Safety

Adverse drug reactions (ADRs) are a significant cause of iatrogenic morbidity, mortality, and high cost [69]. They are one of the most common causes of death [70]. Today PGx is not a routine in clinical practice which may explain the lack of statistically significant data about the underlying reasons for ADRs-caused mortality. However, it is well known that the majority of ADRs are dose-dependent while the rest are related to allergy or idiosyncratic [71]. Usage of anticoagulants, opioids, or immunosuppressants is the most frequent reason for the lethal outcome [71]. At the same time, lethality indicator strictly correlates with the age, race, and urbanization level [72,73,74,75].

ADRs can result from inappropriate drug prescription, toxic effects of drug chemicals, impaired absorption, distribution, metabolism, and elimination of drugs related to age and sex, drug-drug interactions in combination therapy, or when a patient is treated with different medications for comorbid disorders [70]. This is especially important in chronic diseases that require long-term treatment and the treatment of the elderly, who, in 50% of cases or more, take several types of drugs daily [70]. To mitigate side effects, a large number of drug information resources and drug interactions have been developed over the past two decades to provide support to clinicians in making appropriate drug prescription decisions [70]. However, few resources include PGx as a practical tool for clinical use [70].

It is essential to develop interventional approaches to identify patients at risk of side effects to achieve favorable treatment results. Although the risk of developing ADRs may depend on clinical characteristics (organ functions of patients, their age, or the use of potentially interacting drugs), up to 10–20% of ADRs can be caused by genetic factors [69]. For example, genetic polymorphism can lead to metabolic disorders, which leads to the accumulation of drugs or toxic metabolites, and as a consequence to immune-mediated ADRs that can be potentially fatal [69].

The study [44,76,77] showed that women are more receptive to the ADRs. This may be related to gender differences in the pharmacokinetics and pharmacodynamics of drugs [78]. Also, the incidence of ADRs is higher in elderly patients, which has also been confirmed by other studies [79,80]. 

It is quite challenging to control and manage the development of ADRs. The occurrence of ADRs can increase treatment costs due to an increase in the period of hospitalization and additional clinical trials. In addition, ADRs can often lead to cascading processes where new drugs are prescribed for conditions that result from the use of another drug. Although some of the side effects are considered non-preventable, recent developments show that these reactions can be avoided by individualizing drug therapy based on genetic information obtained from pharmacological testing. For example, dihydropyrimidine dehydrogenase (DPD) was an enzyme involved in the detoxification of 5-fluorouracil, a crucial anti-cancer agent. Studies have shown that an inherited DPD defect can lead to severe toxicity associated with 5-fluorouracil, such as myelotoxicity, gastrointestinal toxicity, and neurotoxicity in cancer patients [81]. 

In the treatment of HIV, the drug Abacavir is prescribed, and one of the ADRs is hypersensitivity syndrome (HSS) [69]. HSS leads to systemic disease that manifests itself as fever and maculopapular rash. ADRs usually disappear after discontinuation of Abacavir, but can also be fatal if, despite the response, the drug is continued.

The pharmacogenetic test HLA-B * 57: 01 administered before the initiation of Abacavir effectively eliminated the HSS previously observed in approximately 5% of the treated European population [69].

The effectiveness of the HLA-B * 57: 01 test is explained by its high negative predictive value [82]. Patients lacking regular alleles are more suspicious to develop immunological hypersensitivity to Abacavir, strong evidence of clinical efficacy [83], and cost-effectiveness [84].

## 5. Obstacles on the Way to the Introduction of Pharmacogenetic Tests into Clinical Practice

PGx testing aims to personalize drug therapy to improve the effectiveness of drug prescription and minimize adverse effects. Despite the potential benefits, PGx testing applications are limited mainly to the use in specialized medical centers or laboratories. The large-scale dissemination and implementation of PGx tests in the typical laboratory and clinical practice are limited by several problems, including legal and ethical issues, scarce data on the effectiveness, validity, and prospects of clinical use, provision of hands-on training for clinicians, testing simplicity and the availability of alternative methods for drug reactions prediction [85,86].

Despite the PGx test constitutes a relatively new approach requiring additional investigations to be introduced in clinical practice population-wide, many companies actively develop in this direction (Table 2).

However, it should be kept in mind that the integration of PGx tests into clinical practice is primarily determined by the fact that the test’s value and relevance depend on whether the test improves clinical outcomes, e.g., decreases morbidity, mortality, and ADRs [87,88].

The 2000 ACCE (analytical validity, clinical validity, clinical utility, and associated ethical, legal and social implications) project by the US Office of Public Health Genomics (OPHG) aims at the evaluation of genetic tests in the Centers for Disease Control and Prevention [89].

The sensitivity and specificity of the test determine the analytical and clinical validity of PGx testing. The analytical validity measures the ability of a test to identify the genotype of interest. At the same time, the clinical one determines the strength of the relationship between the genotype and the endpoint. Test results provide the data on the clinical utility. The latter improves the clinical outcomes and increases the testing value—compared to the absence of a test or standard treatment [89,90,91].

Later, the OPHG assigned the Evaluation of Genomic Applications in Practice and Prevention (EGAPP) working group, which expanded and refined the ACCE model. EGAPP supports the development of the systematic assessment of available data regarding the validity of genetic tests and their usefulness in clinical practice, works out recommendations for healthcare professionals [92], and evaluates the widely used genetic tests [93]. The EGAPP Working Group has developed three evidence-based guidelines for PGx tests: (1) genotyping of UGT1A1 for the prediction of response to irinotecan therapy in metastatic colorectal cancer (mCRC), (2) testing tumor tissue on EGFR persistence to choose anti-EGFR therapy for mCRC patients, (3) testing for cytochrome (CYP) P450 polymorphism in adults, suffering from non-psychotic depression, for the prediction of response to selective inhibitors of serotonin reuptake. As a rule, guidelines to these tests indicate insufficient evidence in favor of (or against) doing the test. CPIC develops guidelines for clinicians to help them understand how to use the results of genetic testing for improvement of the drug therapy effectiveness and acceleration of the pharmacogenetic knowledge uptake as a result [94].

The reproducibility and reliability of the data obtained represent a common problem of pharmacological testing [14]. The clinical validity of research can be determined by classical methods such as meta-analysis [95], which represents a systematic review of the literature to evaluate and synthesize all available data on a specific issue [96]. A meta-analysis demonstrated that individual or pioneer studies could provide inconsistent or contradictory results [31]. At the same time, research collaborations allow one to combine the results and to consider all available data (both non-published and published) in the meta-analysis. This provides a larger sample size, yielding a more accurate estimation of the association [97]. However, summarizing and processing large amounts of data can be very complicated and time-consuming [98].

Another problem limiting the PGx test integration into practice is the lack of (or insufficient) evidence to support the usefulness of the test. Randomized controlled trials (RCTs) usually give compelling evidence that preventive and predictive testing improves clinical outcomes with drug therapy. However, if the influence of the genotype is insignificant or the pathological condition is rare, an RCT may require extensive samples, engaged for decades or more [14,99]. In addition, RCTs are expensive, thus being difficult to find funding sources for, especially for low-cost or generic drugs [100]. The PGx test design also represents another restraint for using RCTs: the tests involve a limited number of polymorphisms in specific candidate genes. For instance, the response to depression drugs has a very polygenic architecture [101]. Each polymorphism is assumed to result in a 2–3% variance in the response [102]. Another problem is that the test should take into account polymorphism interaction. The most recent reviews indicate that published studies provided limited cost/benefit data for the available PGx tests. In some cases, the studies found limited (or the absence of) clinical benefit (improved response or remission, decreased adverse effects) [103,104,105].

There is certainly controversy about the evidence which will be reliable and, at the same time, really feasible to identify the PGx tests’ clinical usefulness [91,106]. Many authors believe that a combination of retrospective and prospective studies will do the job; however, the recommendations will require considering the limitations of each research method [86,90].

In addition to scientific evidence, guidelines, and regulations developed, healthcare workers’ willingness and ability to use the proposed tests determine the PGx tests scale-up [14,107]. Some time ago, healthcare professionals were reluctant to accept pharmacogenetics, although this position may have changed over time [108]. It may be difficult for specialists to change the tactics of treatment using the usual drugs since they already have a practice of leveling the side effects of the drugs used [109,110].

Most clinicians still lack confidence in PGx testing and subsequent data interpretation, indicating insufficient knowledge in this field [86,90]. The literature emphasizes the need to improve literacy among healthcare professionals regarding expertise in and understanding of PGx testing [14,111].

Lack of awareness of practitioners about the possibilities of pharmacogenetics and poor or insufficient explanation of the test results also reduce personalization technologies for patients. In addition to the development of thematic training courses at medical universities, the inclusion of educational cycles in continuing professional education systems, free placement of information for practicing doctors are required: educational internet portals, webinars, etc. A clinical pharmacologist plays a crucial role in the implementation of pharmacogenetic testing. The competence of a clinical pharmacologist in the field of pharmacogenetics is critical: he or she is the one who organizes the application of genotyping in clinical practice, interprets tests, informs doctors about the possibilities of pharmacogenetics for patients with specific nosologies, that is, acts as the main link between the scientific world, the healthcare system and practicing physicians in the process of introducing pharmacogenetics [14,112].

Currently, algorithms for the interpretation of the results of pharmacogenetic testing are mentioned in the

- Instructions for the medical use of a medicinal product (FDA and European Medicines Agency (EMA)), recommendations of international and national professional scientific public organizations (Recommendations of the experts of the European Science Foundation (ESF), discussed and approved by the participants European Conference on Pharmacogenetics and Pharmacogenomics in Barcelona in June 2010 (published in March 2011) [113],
- Expert recommendations of the Pharmacogenetics Working Group of the Royal Dutch Pharmaceutical Association (published in March 2011) [50],
- Expert guidance of the CPIC, beginning of publication—January 2011) [104].

Disclosure of genetic information to insurance companies is considered an essential socioeconomic issue. This may lead to an increase in health insurance rates for certain patient groups [114]. This problem is deemed to be ethical. It is necessary to determine the group of people who have access to the results of genotyping and formulate the possible consequences for the patient.

The cost of pharmacogenetic testing is another unresolved issue. Even though there are positive results of pharmacoeconomic studies, where the use of pharmacogenetic testing made it possible to reduce the cost of treatment by reducing the cost of correcting the consequences of therapy ineffectiveness or unwanted ADRs, not all insurance companies and health systems are ready to include genotyping in their programs. The cost of genetic tests is decreasing every year, but testing is still available to a wide range of patients with an average income [115].

## 6. Conclusions

We are on the verge of a new era in human genetic analysis. Deciphering the human genome, together with the development of high-throughput genetic analysis methods, provided a unique opportunity for the identification of complex genetic changes, resulting in the development of new branches of pharmacy: pharmacogenetics and pharmacogenomics. The genomic profiling of patients became a new diagnostic tool, enabling personalized drug therapy with higher effectiveness and fewer adverse effects. The pharmacogenetic tests can help select a specific drug with a specific dosage and administration regime, which will meet the requirements for the treatment of a particular patient in a specific setting, allowing one to avoid time-consuming dose adjustment inevitably associated with adverse effects.

Although PGx testing represents a significant therapeutic advance, it is just a healthcare professional’s arsenal tool of the future, increasing therapy effectiveness. The technological breakthrough will never override a physician’s experience and logic. This is a small paradigm shift towards the personalization of treatment. 

PGx testing development inevitably will stimulate the progress in methods of data storage and data analysis, which are required for the integration of modern information technologies into routine clinical practice. However, the improvement in digital technologies, the increase in the volume and accessibility of databases is not the only problem in integrating genetic testing into the healthcare continuum. This, in turn, requires changes in the interaction between an individual patient and the healthcare system since the ultimate goal is the patient’s recovery or control of the disease, regardless of what laboratory techniques and data analysis technologies are employed. In other words, the treatment should take into account both the patient’s requirements and the test results. Today pharmacogenetics is in its infancy. A large pool of experimental, but mostly pilot, studies in this area have been accumulated; however, this information can barely be classified as systemic, which makes it difficult to explain the observed correlations between the presence of polymorphisms of a separate gene and epigenetic factors, or the severity of the course of the disease/resistance to therapy. The authors do not aspect information on congenital/acquired polymorphisms, even for the genetically determined diseases, such as cardiovascular diseases and diabetes mellitus. So far, pharmacogenetics provides mosaic information related to the association between the response to drug therapy depending on the genetic background. The next stage is expected to be the research on a larger group of participants, study the contribution of epigenetic factors, and providing clinical guidelines for adjusting or selecting the therapy based on the personal characteristics of the patient. Nevertheless, the field of pharmacogenetics is being actively developed and discussed, and the current demand for end-products in medicine is unusually high. We expect that soon researchers find answers to many questions that are still controversial.

## Figures and Tables

**Table 1:** The most visited and popular internet resources in the pharmacogenetic sector.

Resource	Description	Reference
Coursera	Online personalized medicine course that provides short educational courses in genetics and mechanisms determining the variability of response to drugs; development of ethical issues and objections related to implementation and introduction of wide-scale genome-sequencing into clinical practice.	https://www.coursera.org/learn/personalizedmed
CPIC	An international consortium that specializes in publishing genotype-based drug guidelines to help clinicians understand the usability of the available genetic test results in optimizing drug therapy.	https://www.cpicpgx.org [50,51]
eMERGE	Funded by the NIH. This network brings together researchers with a wide range of expertise in genomics, statistics, ethics, informatics, and clinical medicine from leading medical research institutions across the country to research in genomics including the discovery, clinical implementation, and public resources.	https://www.emerge-network.org
GTR	Free of charge resource that provides generalized datastore of the exhaustive information about genetic tests which is provided and supported by vendors; main auditory is clinicians and researchers.	https://www.ncbi.nlm.nih.gov/gtr [52]
IGNITE	Was developed to enhance the use of genomic medicine by supporting the incorporation of genomic information into clinical practice and exploring methods for effective implementation, diffusion, and sustainability in various clinical settings.	https://www.gmkb.org
My Drug Genome	A portal to study how genetics affects drug response and how results of genetic testing can be implemented into healthcare.	https://www.mydruggenome.org
PharmGKB	Online knowledge base responsible for the aggregation, curation, integration, and distribution of data on the influence of genetic variation on the drug response in humans.	https://www.pharmgkb.org [53]

CPIC—Clinical Pharmacogenetics Implementation Consortium; eMERGE—Electronic Medical Records and Genomics Network; GTR—Genetic Testing Registry; IGNITE—GeNomics In pracTicE; NIH—National Institutes of Health; PharmGKB—Pharmacogenetic Knowledge Base.

**Table 2:** Pharmacogenomic companies and services.

Companies	Main Activity	Reference
Ayass BioScience	A disease monitoring system for implementation in modern molecular medicine and daily clinical practice. Emphasizes genetic, epigenetic, proteomic, and metabolomic profiling, for data collection and interpretation using bioinformatics and biostatistics.	https://ayassbioscience.com
Biocerna	This company elaborated a PGx360™ test which is a panel of 22 genes and 62 associated variants to provide an opportunity for clinicians in the selecting of a proper drug. “Biocerna” also provides specific testing of translocations used to monitor a patient’s response to chemotherapy strategy.	http://www.biocerna.com
Coriell Life Science (Gene Dose)	Provides data analysis of and reports on clinical laboratory pharmacogenomic assays. Coriell Life Sciences PGx elucidates results of PGx assays in association with drug-related risks to improve patient health to provide a complete, safe, and personalized drug therapy strategy.	https://www.coriell.com
Diatech Pharmacogenetics	Develops pharmacogenetic tests for precision cancer medicine and produces two groups of cancer therapy products: (1) pyrosequencing technology in pharmacogenetics of anti-EGFR therapy, and (2) pyrosequencing technology in pharmacogenetics of chemo- and radiotherapy.	https://www.diatechpharmacogenetics.com
Dynamic DNA Laboratories	This company provides a wide variety of gene testing services, including pharmacogenomic testing, drug discovery, DNA expression, and also some customized testing and DNA testing services. The main PGx product is the predictive Comprehensive PGx Test for over 150 different drugs.	https://dynamicdnalabs.com
Eurofins Genomics	Leader in food, environmental, pharmaceutical, and cosmetic testing. Specializes in pharmacogenetics and PGx research, offers a comprehensive package of services for the drug development process.	https://www.eurofins.com
Exceltox Laboratories	A CAP and CLIA accredited laboratory that offers advanced clinical, PGx, and toxicological analysis.	http://exceltox.com
GeneDx	Leader in genomics, including research on rare genetic diseases. PharmacoDx targets sequence variants in genes that contribute to drug metabolism. PharmacoDx is a comprehensive pharmacogenetic panel with over 100 genetic variants.	https://www.genedx.com
Genentech	A biotechnology company that pioneers research in and develops medications for patients with severe and life-threatening diseases.	https://www.gene.com
Genewiz	A leading international company that offers a wide range of services in genomic technologies, including NGS, classic Sanger sequencing, elaboration of synthetic genes, and bioinformatic data analysis support.	https://www.genewiz.com/en-GB
HudsonAlpha Institute for Biotechnology	Initiation and support of scientific research programs related to human health and well-being; supports the introduction of genomic medicine into clinical practice and promotes entrepreneurship in life sciences. Developing of work programs for specialists in genomics. Conducting extensive elaboration in the pharmacogenetic testing platform in collaboration with Kailos Genetics.	https://hudsonalpha.org
Integrated DNA Technologies	Develops and manufactures nucleic acid products. Areas of activity include scientific and commercial research, agriculture, medical diagnostics, pharmaceutical development, and synthetic biology.	https://www.idtdna.com
Myriad Genetics	Pioneering researches and innovations in molecular diagnostic testing aimed to improve personalized medicine.	https://myriad.com
Pathway Genomics	A company private that offers customized tests for the screening of diet, weight loss, and metabolic response to numerous commonly prescribed medications. The information can be securely delivered to patients and physicians through any mobile device in a comprehensive form using the in-house developed application. The company produces several PGx products, including “OmePsychiatricMeds” (genetic test for mental health medication efficacy) and “OmePainMeds” (genetic test for pain management medication efficacy).	https://www.pathway.com/about
Phenomics Health	This is a bioinformatic platform for precision medicine that transforms large health data sets of patients and even populations into certain products and services to support decision-making about pharmacological treatment.	https://www.phenomicshealth.com/
Quantigen	Development of gene expression and gene variation tests, methods validation, and other services related to clinical assays and PGx researches.	http://www.quantigen.com
RxGenomix	Developed a new highly secured and compatible data-concentrator RxGenomix that provides genomic data exchange through distinct healthcare IT services including laboratory management systems, electronic clinical records, and pharmacy operation systems.	https://www.rxgenomix.com
Sema4	This is an interdisciplinary partnership of scientists, clinicians, engineers, and genetic consultants. It is a unique consortium with a solid basis of more than 160 years of clinical experience, world-class academic research, and groundbreaking information technology.	https://sema4.com
Sorenson Genomics	This company is mainly focused on DNA testing in forensic and research projects. The main areas of interest are DNA genotyping, DNA sequencing and analysis of fragments in population genetics, and human genotyping. Offers: LEAD Local Entry Access DNA Database—a reliable, proven software that allows fast and secure storage, search, and analysis of millions of DNA profiles.	https://sorensongenomics.com
Transgenomic	Development of molecular technologies for personalized medicine specifically in cardiology and oncology. This company is an international leader in pharmacogenetic testing and offers a variety of products designed to detect specific mutations in a certain gene that can indicate a specific heart disease and risk of heart failure.	http://www.transgenomic.com
Translational Software	A leader in the use of genetic data purposed to support decision-making in precision medicine. Developed software that enables laboratories and clinicians to incorporate PGx data into treatment strategies to improve a personalized approach such as “PGxAPI”—a knowledge base purposed to include PGx data into healthcare and laboratory systems. PGxPortal offers an HL7 interface for receiving data and checking the quality of test results. This portal enables clinicians to deliver better patient care by providing clinically relevant pharmacogenomic information.	https://www.translationalsoftware.com
Xact Laboratories	A molecular diagnostics laboratory with a sophisticated research approach that provides a wide range of custom-centered distinctive tests for clinicians and healthcare providers.	https://xactlaboratories.com
23andMe	A biotechnology company that provides customers with information on their disease susceptibility. Pharmacogenetic studies include analysis of CYP2C19, DPYD, SLCO1B1.	https://www.23andme.com

CAP—College of American Pathologists; CLIA—Clinical Laboratory Improvement Amendments; CYP2C19—cytochrome P450 2C19; DPYD—dihydropyrimidine dehydrogenase; SLCO1B1—solute carrier organic anion transporter family member 1B1.
